# Different MDSC Activity of G-CSF/Dexamethasone Mobilized Neutrophils: Benefits to the Patient?

**DOI:** 10.3389/fonc.2020.01110

**Published:** 2020-07-21

**Authors:** Cathelijn E. M. Aarts, Ida H. Hiemstra, Charita Furumaya, Robin van Bruggen, Taco W. Kuijpers

**Affiliations:** ^1^Department of Blood Cell Research, Sanquin Research, Amsterdam University Medical Center (AUMC), University of Amsterdam, Amsterdam, Netherlands; ^2^Department of Pediatric Immunology, Rheumatology & Infectious Diseases, Emma Children's Hospital, AUMC, University of Amsterdam, Amsterdam, Netherlands

**Keywords:** neutrophils, MDSC activity, granulocyte transfusions, GTXs, mobilized-neutrophils

## Abstract

Human neutrophils exert a well-known role as efficient effector cells to kill pathogenic micro-organisms. Apart from their role in innate immunity, neutrophils also have the capacity to suppress T cell-mediated immune responses as so-called granulocyte-myeloid-derived suppressor cells (g-MDSCs), impacting the clinical outcome of various disease settings such as cancer. Patients undergoing chemotherapy because of an underlying malignancy can develop prolonged bone marrow suppression and are prone to serious infections because of severe neutropenia. Concentrates of granulocytes for transfusion (GTX) constitute a therapeutic tool and rescue treatment to fight off these serious bacterial and fungal infections when antimicrobial therapy is ineffective. GTX neutrophils are mobilized by overnight G-CSF and/or Dexamethasone stimulation of healthy donors. Although the phenotype of these mobilized neutrophils differs from the circulating neutrophils under normal conditions, their anti-microbial function is still intact. In contrast to the unaltered antimicrobial effector functions, G-CSF/Dexamethasone-mobilized neutrophils were found to lack suppression of the T cell proliferation, whereas G-CSF-mobilized or Dexamethasone-mobilized neutrophils could still suppress the T cell proliferation upon cell activation equally well as control neutrophils. Although the mechanism of how G-CSF/Dex mobilization may silence the g-MDSC activity of neutrophils without downregulating the antimicrobial activity is presently unclear, their combined use in patients in the treatment of underlying malignancies may be beneficial—irrespective of the number of circulating neutrophils. These findings also indicate that MDSC activity does not fully overlap with the antimicrobial activity of human neutrophils and offers the opportunity to elucidate the feature(s) unique to their T-cell suppressive activity.

## Introduction

Patients who undergo chemotherapy are prone to develop neutropenia and are thereby susceptible to serious bacterial and fungal infections (1). In addition to antimicrobial therapy, granulocyte transfusions (GTX) can be a therapeutic option to improve the clinical outcome in case of a deteriorating clinical condition because of the lack of efficacy of antimicrobial agents only (2–4). In the past we and others have extensively described the combined administration of G-CSF and Dexamethasone to healthy donors in order to generate sufficient numbers of cells for these GTX products. These mobilized GTX neutrophils show a changed phenotype but a completely intact ability to respond, migrate and kill invading pathogens (5).

Next to their role of efficient innate immunity killers of micro-organisms, neutrophils are also recognized to be involved in modulation of adaptive immune responses in various disease settings including cancer (6–9). Immature and mature neutrophils were reported to have the capacity to suppress T cell-mediated immune responses as so-called granulocyte-myeloid-derived suppressor cells (g-MDSCs), and thereby affect the clinical outcome of cancer patients. In fact, in cancer patients the presence of increased neutrophil counts in the circulation is directly related with a bad prognosis (9). While the function of g-MDSCs has been investigated in depth and in murine experimental models in particular, the characterization of human g-MDSC activity is still controversial. Lectin-type Oxidized LDL receptor 1 (LOX-1) has been suggested to be a marker to discriminate g-MDSCs from circulating human mature neutrophils and would therefore allow for better distinction without the use of a gradient (low-density g-MDSCs versus high-density mature neutrophils) (10). However we have found in a recent study that activated mature neutrophils also express LOX-1 (11), questioning the fact if LOX-1 is indeed a suitable g-MDSC marker. We have recently demonstrated that mature neutrophils (i.e., high-density) from healthy donors can exert MDSC activity (i.e., suppress immune responses) but only upon cell activation (11–13), which correlates to the LOX-1 expression. Moreover, the mechanisms involved in the MDSC activity greatly overlapped with the toxic antimicrobial effector functions of neutrophils, being dependent on cell-cell contact (adhesion), production of reactive oxygen species (ROS) and release of their granular content (degranulation) (11).

In this study we investigated whether neutrophils obtained upon overnight mobilization of neutrophils into the bloodstream in healthy GTX donors may have a potentially relevant impact as MDSCs in the treatment of oncology patients.

## Materials and Methods

### Study Approval

Heparinized peripheral blood samples were collected from healthy granulocyte transfusion donors 1 day after combined G-CSF (600 μg, subcutaneously) and dexamethasone (8 mg, orally) treatment (G-CSF/Dex), or upon their preference with G-CSF or dexamethasone alone, as described previously (5). Blood samples were collected after obtaining informed consent and in accordance with the Declaration of Helsinki.

### Blood Cell Isolation

Neutrophils and peripheral blood mononuclear cells (PBMC) were isolated from whole blood by gradient centrifugation using isotonic Percoll (Pharmacia, Uppsala, Sweden) with a specific density of 1.076 g/mL. T cells were isolated from the PBMC fraction by magnetic-activated cell sorting with the Pan T cell isolation kit of Miltenyi-Biotec (Bergisch Gladbach, Germany) according to the manufacturer's instructions. Neutrophils were obtained from the pellet fraction after erythrocyte lysis with hypotonic ammonium chloride solution at 4°C as previously described (14).

### T Cell Proliferation Assay

Purified T cells were labeled with CFSE (Molecular probes, Life Technologies, Carlsbad, CA, USA) and cultured in 96-well flat bottom plates (Nunclon Delta Surface, Thermo Scientific, Waltham, MA, USA) for 4–6 days at 37°C in IMDM medium (Gibco, Life Technologies, Carlsbad, CA, USA), supplemented with 10% (v/v) fetal calf serum (Bodinco, Alkmaar, The Netherlands), 10^4^ U/mL penicillin, 10 ng/mL streptomycin, 200 mM glutamine, and 0.00035% (v/v) β-mercaptoethanol (Sigma-Aldrich, Saint Louis, MO, USA). To induce proliferation, the T cells were stimulated by anti-CD3 (clone 1XE [IgE isotype] hybridoma supernatant, 1:1,000, Sanquin, Amsterdam, The Netherlands) and anti-CD28 (clone 15E8 [IgG1 isotype] at 5 μg/mL, Sanquin) monoclonal antibodies (moAbs; at 20,000 T cells/well). Neutrophils from blood, collected from the pellet fraction after density centrifugation, were added in a 1:3 ratio (60,000 neutrophils/well), in the presence or absence of neutrophil-activating stimuli: fMLF (1 μM, Sigma), TNFα (10 ng/mL, Peprotech EC, London, UK) or LPS (20 ng/mL, *E. coli* 055:B5, Sigma).

After 4–6 days, the cells were harvested from the culture plates and stained with APC-labeled anti-CD4 (clone SK3, BD Biosciences, San Jose, CA, USA) and PerCPCy5.5-labeled anti-CD8 (clone SK1, Biolegend, San Diego, CA, USA) antibodies. The T cell proliferation was assessed by measuring the CFSE dilution of CD4^+^ and CD8^+^ T cells via flow cytometry.

### ROS Production

NADPH oxidase activity was assessed as the release of hydrogen peroxide, determined by the Amplex Red method (Molecular Probes, Life Technologies, Carlsbad, CA, USA) by neutrophils (1x10^6^/mL) stimulated with: fMLF (1 μM), TNFα (10 ng/mL), LPS (20 ng/mL) + LPS-binding protein (LBP) (50 ng/mL, R&D Systems, Minneapolis, MN, USA) or PMA (100 ng/mL, Sigma) in the presence of Amplex Red (0.5 μM) and horseradish peroxidase (1 U/mL). Fluorescence was measured at 30-s intervals for 4 h with the HTS7000+ plate reader (Tecan, Zurich, Switzerland). Maximal slope of hydrogen peroxide release was assessed over a 2-min interval.

### Antibodies and Flow Cytometry

The following directly conjugated antibodies were used for flow cytometry analysis: PB-labeled anti-CD11b (clone ICRF44, BD Biosciences) and PECy7-labeled anti-CD16 (clone 3G8, BD Biosciences).

Flow cytometry data were acquired using Canto II flow cytometer (BD Biosciences) and analyzed using FlowJo software (Tree Star, USA).

### Statistics

Statistical analysis was performed with GraphPad Prism version 8 for Windows (GraphPad Software, San Diego, CA, USA). Data were evaluated by one-way ANOVA or unpaired two-tailed student's *t*-test. The results are presented as the mean ± SEM. Data were considered significant when *p* < 0.05.

## Results

### G-CSF/Dex Mobilized Neutrophils Are Not Able to Suppress the T Cell Proliferation

We received blood from healthy granulocyte transfusion donors routinely treated with the combination of G-CSF and dexamethasone to test whether the mobilization of neutrophils into the bloodstream resulted in a change of MDSC activity.

**Figure 1:**
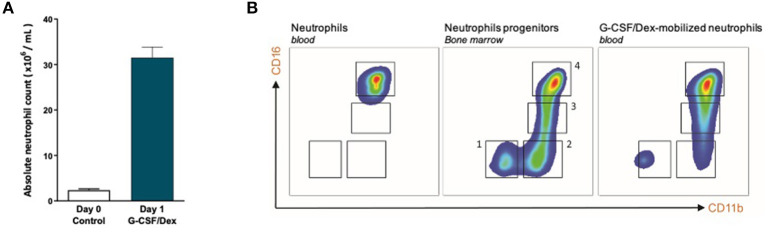
G-CSF/Dex-mobilized donors have an increased amount of neutrophils including immature and mature neutrophils. **(A)** Absolute neutrophil count of peripheral blood from G-CSF/Dex-treated donors before and after administration *n* = 5. **(B)** Surface marker expression of CD11b and CD16 was measured by flow cytometry analysis of neutrophils from blood of healthy donors (left panel), neutrophil progenitors from bone marrow (center panel) and G-CSF/Dex-mobilized neutrophils. The four indicated neutrophil progenitor populations are (pro)myelocytes (1, CD11b^NEG^CD16^NEG^), metamyelocytes (2, CD11b^POS^CD16^NEG^), band cells (3, CD11b^POS^CD16^DIM^) and segmented cells (4, CD11b^POS^CD16^POS^). Shown are representative FACS analysis images (*n* = 3).

One day after G-CSF/Dex administration, the absolute neutrophil count in the peripheral blood was ~30 times increased compared to the neutrophil count before administration (Figure 1A). The rapid increase in blood neutrophil numbers induced by G-CSF/Dex resulted from the predominant release of mature (~80%) and some immature (~20%) neutrophils from the bone marrow into the circulation (Figure 1B). Neutrophil progenitor cells can be divided in four different developmental stages, namely (pro)myelocytes, metamyelocytes, band cells and segmented neutrophils based on the expression of cell surface markers CD11b and CD16 (15, 16), which were all present in the G-CSF/Dex-mobilized neutrophil fraction (Figure 1B). Apart from the release of the reserve pool of neutrophils from the bone marrow, also the demargination of neutrophils from the (lung) vasculature as well as activation of neutrophils due to the overnight G-CSF/Dex may contribute to a change in phenotype and function of these GTX neutrophils (5). Although the exact contribution of each of these processes remains unclear, G-CSF/Dex-mobilized neutrophils have a completely intact ability to respond to signs of infection, migrate toward an ongoing infection and kill invading pathogens as we had previously studied in great detail (5).

To investigate the MDSC activity (i.e., suppression of immune responses) of these G-CSF/Dex-mobilized neutrophils, we now performed additional T cell proliferation assays. In our previous study (11), where we have optimized our T cell proliferation assay, we have studied the mechanism behind the suppressive activity of activated mature neutrophils in more depth. Here we found that neutrophils exert their suppressive activity in the first hours/day of the cell culture, after which the suppressed T cells are no longer prone to T cell stimulation. Furthermore, the most optimal read-out of the T cell proliferation by CFSE dilution was between 4 and 6 days of cell culture.

Neutrophils from G-CSF/Dex donors or from healthy controls were cultured simultaneously for 5 days in the presence of isolated CFSE-labeled T cells from an unrelated healthy donor and were left unstimulated or activated with either fMLF, TNFα, or LPS. Just as previously described, T cell proliferation was induced by the strong and uniform activation by the combination of monoclonal anti-CD3 and anti-CD28 antibodies and quantified as relative “recursor frequency”: i.e., percentage of naïve cells in the initial population that underwent one or more divisions upon anti-CD3/anti-CD28 antibodies (17). The precursor frequency was then normalized for the condition of anti-CD3/CD28-stimulated T cells and non-activated neutrophils.

**Figure 2:**
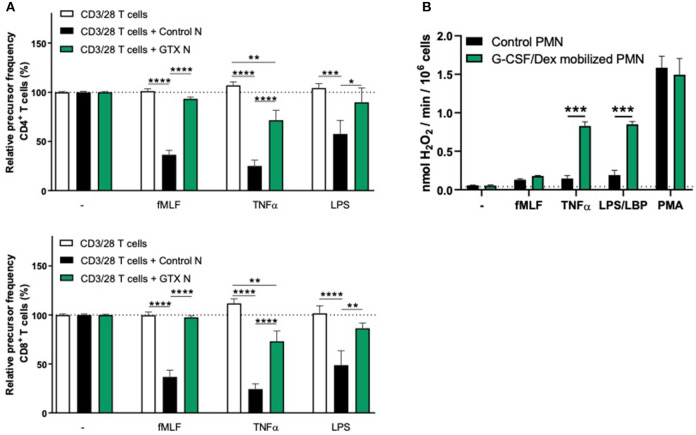
G-CSF/Dex-mobilized neutrophils cannot suppress T cell proliferation despite normal/increased ROS production. **(A)** Purified CFSE-labeled T cells from healthy donors (*n* = 4) were cultured in absence (white bars) or presence of mature neutrophils from control donors (black bars, *n* = 4), or G-CSF/Dex-mobilized neutrophils (green bars, *n* = 3). T cells were stimulated with anti-CD3 and anti-CD28 antibodies. Neutrophils were kept unstimulated or activated with the indicated stimuli. After 4 or 5 days, cells were harvested and analyzed by flow cytometry for CFSE dilution among CD4^+^ (upper graph) and CD8^+^ (lower graph) T cells. Error bars indicate SEM; the statistical analysis one-way ANOVA was used. **(B)** G-CSF/Dex-mobilized neutrophils or control neutrophils were stimulated with the indicated stimuli and production of H_2_O_2_ was determined by measuring Amplex Red conversion into fluorescent Resorufin (*n* = 3–4). Error bars indicate SEM; the statistical analysis unpaired two-tailed *t-*test was used. *p < 0.05, **p < 0.01, ***p < 0.001, ****p < 0.0001.

We observed that G-CSF/Dex-mobilized neutrophils were not able to suppress the T cell proliferation of CD4^+^ or CD8^+^ T cells, neither under resting conditions nor upon their activation (Figure 2A). Only the neutrophils from healthy controls were able to suppress T cell proliferation upon proper activation. One of the main effector mechanism in which activated neutrophils suppress the T cell proliferation is the production of reactive oxygen species (ROS) (11, 18–20). The G-CSF/Dex-mobilized neutrophils showed normal ROS production upon fMLF stimulation and even to a larger extent upon TNFα or LPS/LBP stimulation, when compared to neutrophils from healthy donors (Figure 2B). These data indicate that the lack of MDSC activity of G-CSF/Dex-mobilized neutrophils cannot be ascribed to an impaired respiratory burst. As previously shown, also degranulation and adhesion properties were unremarkable and very similar to those of normal neutrophil from healthy controls without prior mobilization for GTX products (5, 21).

### Both G-CSF- and Dex-Mobilized Neutrophils Can Suppress the T Cell Proliferation

**Figure 3:**
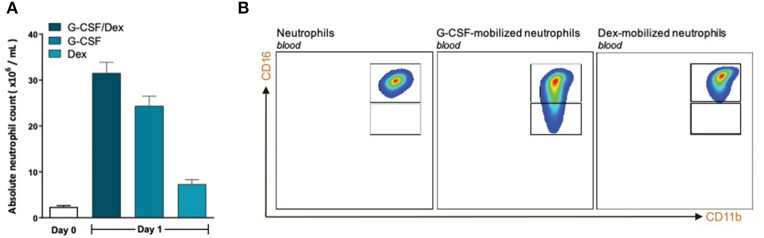
G-CSF- and Dex-mobilized donors have an increased amount of neutrophils consisting mostly of mature neutrophils. **(A)** Absolute neutrophil count of peripheral blood of healthy donors (white bar, *n* = 6) or from G-CSF/Dex-treated, G-CSF-treated or Dex/treated donors 1 day after administration (*n* = 3). **(B)** Surface marker expression of CD11b and CD16 was measured by flow cytometry analysis of neutrophils from blood of healthy donors (left panel), G-CSF-mobilized neutrophils (center panel) and Dex-mobilized neutrophils. Shown are representative FACS analysis images (*n* = 3).

**Figure 4:**
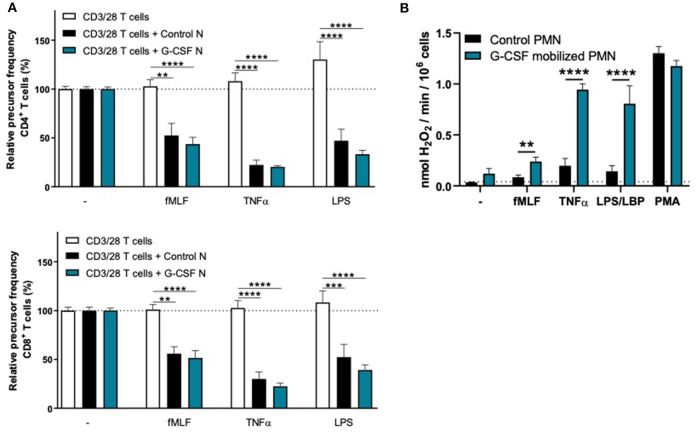
G-CSF-mobilized neutrophils can suppress the T cell proliferation and have an elevated respiratory burst. **(A)** Purified CFSE-labeled T cells from healthy donors (*n* = 6) were cultured in absence (white bars) or presence of mature neutrophils from control donors (black bars, *n* = 6), or G-CSF-mobilized neutrophils (blue bars, *n* = 3). T cells were stimulated with anti-CD3 and anti-CD28 ntibodies. Neutrophils were kept unstimulated or activated with the indicated stimuli. After 4 or 5 days, cells were harvested and analyzed by flow cytometry for CFSE dilution among CD4^+^ (upper graph) and CD8^+^ (lower graph) T cells. Error bars indicate SEM; the statistical analysis one-way ANOVA was used. **(B)** G-CSF-mobilized neutrophils or control neutrophils were stimulated with the indicated stimuli and production of H_2_O_2_ was determined by measuring Amplex Red conversion into fluorescent Resorufin (*n* = 3–6). Error bars indicate SEM; the statistical analysis unpaired two-tailed *t-*test was used. **p < 0.01, ***p < 0.001, ****p < 0.0001.

**Figure 5:**
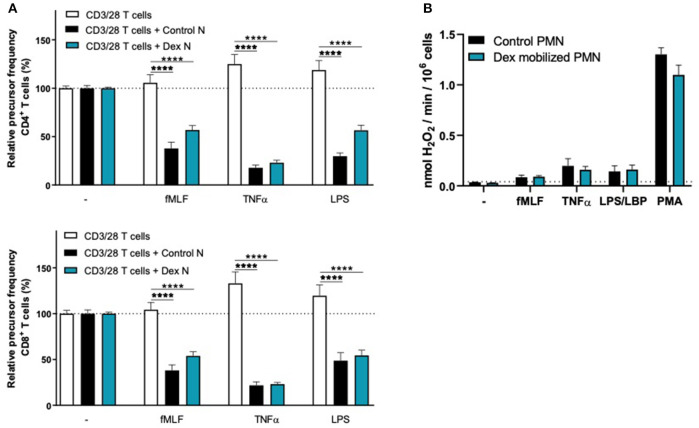
Dex-mobilized neutrophils can suppress the T cell proliferation and have normal respiratory burst. **(A)** Purified CFSE-labeled T cells from healthy donors (*n* = 4) were cultured in absence (white bars) or presence of mature neutrophils from control donors (black bars, *n* = 4), or Dex-mobilized neutrophils (blue bars, *n* = 3). T cells were stimulated with anti-CD3 and anti-CD28 antibodies. Neutrophils were kept unstimulated or activated with the indicated stimuli. After 4 or 5 days, cells were harvested and analyzed by flow cytometry for CFSE dilution among CD4^+^ (upper graph) and CD8^+^ (lower graph) T cells. Error bars indicate SEM; the statistical analysis one-way ANOVA was used. **(B)** Dex-mobilized neutrophils or control neutrophils were stimulated with the indicated stimuli and production of H_2_O_2_ was determined by measuring Amplex Red conversion into fluorescent Resorufin (*n* = 3–6). Error bars indicate SEM; the statistical analysis unpaired two-tailed *t-*test was used. ****p < 0.0001.

To investigate whether the lack of MDSC activity by G-CSF/Dex-mobilized neutrophils is caused by the G-CSF or Dex component, we isolated neutrophils 1 day after administration from healthy donors who had received only G-CSF or dexamethasone. As we had observed with the G-CSF/Dex donors, the absolute neutrophil counts of either G-CSF- or Dex-treated donors were increased in the peripheral blood compared to the numbers of circulating neutrophils prior to the administration of mobilizing agent, i.e., around nine and three times higher, respectively (Figure 3A). Although the increase in circulating neutrophils was not as high as in G-CSF/Dex-treated donors, also the number of immature neutrophils released into the blood stream were lower in case of the use of G-CSF or Dex only. A small population of CD11b^POS^ CD16^DIM^ cells was present in the G-CSF-mobilized neutrophil fraction next to the mature neutrophils (CD11b^POS^ CD16^POS^), whereas the Dex-mobilized neutrophil fraction only comprised phenotypically mature neutrophils (Figure 3B). The G-CSF-mobilized and Dex-mobilized neutrophils were both able to suppress the T cell proliferation of CD4^+^ and CD8^+^ T cells upon activation, comparable to neutrophils from healthy controls (Figures 4A, 5A). Also the activation of the NADPH oxidase complex required for ROS production was intact. Whereas, the G-CSF-mobilized neutrophils showed a higher level of ROS production upon fMLF, TNFα, or LPS/LBP stimulation (Figure 4B), similar to G-CSF/Dex-mobilized neutrophils (Figure 2B). Dex-mobilized neutrophils showed a normal ROS production, comparable to neutrophils from non-mobilized healthy donors (Figure 5B).

Collectively our data suggest that the MDSC activity is only absent when neutrophils are mobilized with both G-CSF and Dexamethasone.

## Discussion

Myeloid-derived suppressor cells have been described as a heterogeneous subset of immature myeloid cells, defined by their capacity to suppress T cell activation and proliferation. Apart from their malignant transformation, tumor cells also create a chronic state of inflammation. In cancer, aberrant emergency myelopoiesis, which is defined as the early exit of progenitor neutrophils from bone marrow, is driven by tumor cell-derived and/or locally tissue-induced factors including colony stimulating factors such as GM-CSF, G-CSF and M-CSF (22, 23). These factors are thought to contribute to the release of immature neutrophil-like cells that represent a unique immature g-MDSC subpopulation (22). The presence and tumor infiltration of MDSCs have been associated with poor prognosis (24–26). Hence, an important issue was raised as to whether treating cancer patients with G-CSF for neutropenia could affect the patients negatively in terms of g-MDSC enrichment (22, 27). However, as we have recently reported by studying bone marrow fractions of myeloid cells in different stages of their development, immature neutrophils are not capable of producing ROS (28). The formation of these toxic metabolites have been shown in several studies to be one of the main effector mechanisms in the suppression of T cells, i.e., MDSC activity (11, 18–20). In line with these observations, we have previously demonstrated that the immature neutrophils in bone marrow fractions from healthy control individuals were not able to suppress the T cell proliferation upon activation. In contrast, MDSC activity in these bone marrow samples was induced in case of the most mature neutrophils being fully differentiated, as indicated by morphology and expression of surface markers (28). Our previous findings on bone marrow derived myeloid progenitors question the presence of a subset of highly effective granulocyte-related MDSCs that can be released into the circulation to fulfill instantaneously strong T cell suppressive activity in humans (28). Although we cannot exclude the presence of such a bone marrow subset in case of the presence of cancer that may chronically induce the development of such a subset of MDSCs, we could not detect such spontaneously active MDSCs in chemotherapy-naïve patients newly diagnosed with Head-Neck Cancer or Mamma Carcinoma (11). Still, a myeloid progenitor MDSC may be released to “home” to the tumor microenvironment to develop locally in a strong suppressor cell but supportive data are as yet not available to the best of our knowledge.

In our previous study (11), MDSC activity of neutrophils in cancer patients and controls was found to be very similar and depended completely on prior activation. The process of MDSC activity was defined by a the damaged small T cell subset undergoing cell death as indicated by morphological alterations and cellular ATP depletion of the T cells. In this study we have not assessed other suppressive activities than the most relevant function by which MDSC activity is defined, i.e., T cell proliferation. In our previous study (11), g-MDSC activity suppressing the T cell proliferation was found to coincide with the lack of cytokine production, making it less likely that a strong induction of T regulatory cells (Tregs) as additional means of suppressive activity would contribute as a result of the direct MDSC activity *per se*. We have also not extended our studies to possible alternative modes of T cell suppression that might be independent of cell-cell contact and may be based on soluble factors otherwise (29), although such factors have limited impact in our mixed cell culture, as previously demonstrated when kept separated by a permeable filter (11).

The reason underlying the inability of G-CSF/Dex mobilized neutrophils to perform MDSC activity is as yet unclear. We may speculate on the sequential steps of MDSC activity following initial cell-cell-interactions to eliminate T cells by ROS and degranulation, which may be facilitated by trogocytosis, i.e., the uptake of membrane fragments from T cells by activated neutrophils. Neutrophil trogocytosis does occur at an early stage during the multi-step process of exerting its full g-MDSC activity, and may be an initial, necessary but not sufficient step in this process. Neutrophils from chronic granulomatous disease (CGD) patients, unable to generate ROS, do not show MDSC activity while the extent of trogocytosis was indistinguishable from that of control neutrophils (11). The fact that both ROS and degranulation are required while being spared in case of G-CSF/Dex mobilized neutrophils leaves us with an as yet unidentified process that seems to be selectively involved in the initiation of g-MDSC activity.

There is sufficient data to support the active role of neutrophil MDSC activity *in-vivo*, for instance, in the ovarian cancer microenvironment (12). MDSC activity of the neutrophils is actively induced by as yet not fully identified substances within the ascites fluid of these patients. Similar results were obtained when pleural fluid of patients with local metastases were tested (12), supporting the *in-vivo* relevance of neutrophil-mediated MDSC activity. Therefore, G-CSF-mobilized neutrophils could have a pro-tumor response when entering the tumor milieu, as we show here, and treating cancer patients with G-CSF alone for neutropenia may be an important issue to reconsider unless dexamethasone can be used simultaneously to reduce the inherent MDSC activity.

The relevance to further elucidate g-MDSC activity and the mechanism by which the combined use of G-CSF and Dex may selectively silence this activity bares important relevance to the use of checkpoint inhibitors as well as use of tumor-infiltrating lymphocytes (TILs) as novel forms of effective immunotherapy to treat cancer (30–32). In cancer patients the presence of increased neutrophil counts in the circulation is directly related with a poor prognosis (9). Our data show that G-CSF/Dex-mobilized neutrophils lack most of their T cell damaging MDSC activity. Thus, G-CSF/Dex treatment may be a way to silence neutrophils within the tumor environment and thereby protect TILs from local damage, and hence help to improve the development of more effective anti-cancer immunotherapies. Our current studies are focusing on differences in cell-cell contact, signal transduction in both neutrophils and T cells as well as proteomics approaches to find out which toxic mechanisms may be impaired such that T cells may stay unimpaired.

In this study, we explored whether g-MDSC activity of neutrophils can be selectively inhibited when treating cancer, while leaving the effector mechanisms of neutrophils against microbial pathogens unaffected, and show that mature G-CSF/Dex-mobilized neutrophils indeed meet such conditions (5). Although GTX products are rarely used in practice, they can be life-saving. The fact that G-CSF/Dex-mobilized products are without MDSC activity would be an additional positive safety issue for using these products in case they are needed. Moreover, these products may help to clarify the mechanisms in place to modulate g-MDSC activity specifically without downregulating the antimicrobial activity.

## Data Availability Statement

The raw data supporting the conclusions of this article will be made available by the authors, without undue reservation, to any qualified researcher.

## Ethics Statement

The studies involving human participants were reviewed and approved by Sanquin Research Institutional Ethical Committee. The patients/participants provided their written informed consent to participate in this study.

## Author Contributions

TK and CA are the principle investigators, who conceived, and designed the study. CA, IH, and CF performed the experiments. RB contributed to the design of the study. CA and IH initiated many of the experiments and performed the analysis. CA wrote the manuscript together with TK. All authors contributed to the article and approved the submitted version.

## Conflict of Interest

The authors declare that the research was conducted in the absence of any commercial or financial relationships that could be construed as a potential conflict of interest.
